# Temporal Periarteritis in Immunoglobulin G4-Related Disease

**DOI:** 10.7759/cureus.90265

**Published:** 2025-08-17

**Authors:** Sho Isoda, Shogo Shirota, Tadayuki Hashimoto, Kazuki Shigetome, Tomio Suzuki

**Affiliations:** 1 General Medicine, Osaka Medical and Pharmaceutical University Hospital, Takatsuki, JPN; 2 General Medicine, Osaka Medical and Pharmaceutical University, Takatsuki, JPN

**Keywords:** fever of unknown origin, igg4-related disease, jaw claudication, palpation of the temporal arteries, temporal periarteritis

## Abstract

Immunoglobulin G4 (IgG4)-related arteritis/periarteritis commonly involves the aorta, carotid arteries, and coronary arteries but rarely involves the temporal artery. Here, we report a case of IgG4-related periarteritis involving the temporal artery. A 79-year-old man presented with fever, bilateral leg pain, and bilateral jaw claudication. Physical examination revealed the absence of the right temporal artery pulse. Ultrasonography of the right temporal artery showed vessel wall thickening, and biopsy revealed marked infiltration of IgG4-positive plasma cells in the adventitia. Serum IgG4 levels were also elevated, confirming the diagnosis of IgG4-related periarteritis of the temporal artery. His symptoms rapidly improved following the initiation of prednisolone. This case demonstrates that IgG4-related arteritis/periarteritis can affect the temporal artery. Moreover, in patients with fever of unknown origin, evaluation of jaw claudication and palpation of the temporal arteries is essential, even in the absence of headache.

## Introduction

Immunoglobulin G4 (IgG4)-related disease (IgG4-RD) is a systemic disorder characterized by elevated levels of IgG4 in the peripheral blood, infiltrative lymphocyte fibrosis, and the presence of IgG4-positive plasma cells in multiple organs [1]. IgG4-RD can cause arteritis/periarteritis, most commonly affecting the aorta, carotid arteries, common iliac arteries, and coronary arteries [2]. Arteritis/periarteritis of IgG4-RD rarely involves the temporal artery and is usually associated with aneurysms [3-6]. In the four previously reported cases, headache was observed in two cases, whereas fever, jaw claudication, and loss of temporal artery pulsation were not described [3-6]. Herein, we report a case of IgG4-related periarteritis of the temporal artery without aneurysm, presenting with fever and jaw claudication.

## Case presentation

A 79-year-old man with no significant medical history was transferred to our hospital because of bilateral leg pain and fever. His leg pain had started six weeks prior, affecting the thighs and lower legs during walking and at rest. Fever over 38.5℃ has been occurring daily for the past three weeks. Two weeks previously, he was admitted to another hospital because of difficulty walking due to pain. Laboratory analysis revealed leukocytosis (white blood cell count: 17,300/mL), high C-reactive protein level (14.22 mg/dL), and a normal creatine kinase level. Tests for various autoantibodies, including antinuclear antibodies, anti-neutrophil cytoplasmic antibodies, anti-ARS antibodies, anti-TIF-γ antibodies, and anti-Mi-2 antibodies, yielded negative results (Table 1). Positron emission tomography/computed tomography did not detect fluorodeoxyglucose uptake anywhere in the body, including the temporal arteries, aorta, and lower limbs.

**Table 1:** Laboratory test results and reference ranges.

Parameter	Result (unit)	Reference range (unit)
White blood cell count	17.3×10³/μL	3.3–8.6×10³/μL
Hemoglobin	10.0 g/dL	13.7-16.8 g/dL
Platelets	52.7×10⁴/μL	15.8-34.8×10⁴/μL
Aspartate aminotransferase	98 U/L	13-30 U/L
Alanine aminotransferase	62 U/L	10–42 U/L
Gamma-glutamyl transpeptidase	39 U/L	13–64 U/L
Alkaline phosphatase	97 U/L	38–113 U/L
Blood urea nitrogen	13 mg/dL	8.0–20.0 mg/dL
Creatinine	0.69 mg/dL	0.65-1.07 mg/dL
Sodium	136 mEq/L	138–145 mEq/L
Potassium	3.8 mEq/L	3.6–4.8 mEq/L
Chloride	103 mEq/L	101–108 mEq/L
C-reactive protein	14.22 mg/dL	<0.14 mg/dL
Antinuclear antibody	<40	<40
Anti-myeloperoxidase antibody	0.8 U/mL	3.5 U/mL
Anti-proteinase 3 antibody	0.5 U/mL	2.0 U/mL
Anti-ARS antibodies	Negative	<5.0
Anti-TIF-γ antibodies	Negative	<5.0
Anti-Mi-2 antibodies	Negative	<5.0

The patient was transferred to our hospital for further investigation. Thorough history taking revealed for the first time that he had bilateral jaw claudication, which began four weeks prior to admission. No complaints of headache, visual disturbance, or leg muscle weakness were reported. Physical examination revealed no tenderness in the temporal arteries or temporomandibular joints, while the right temporal artery pulse was absent. Tenderness was observed in both thighs and lower legs. Upon examination by an ophthalmologist, ischemic optic neuritis was not indicated.

**Figure 1:**
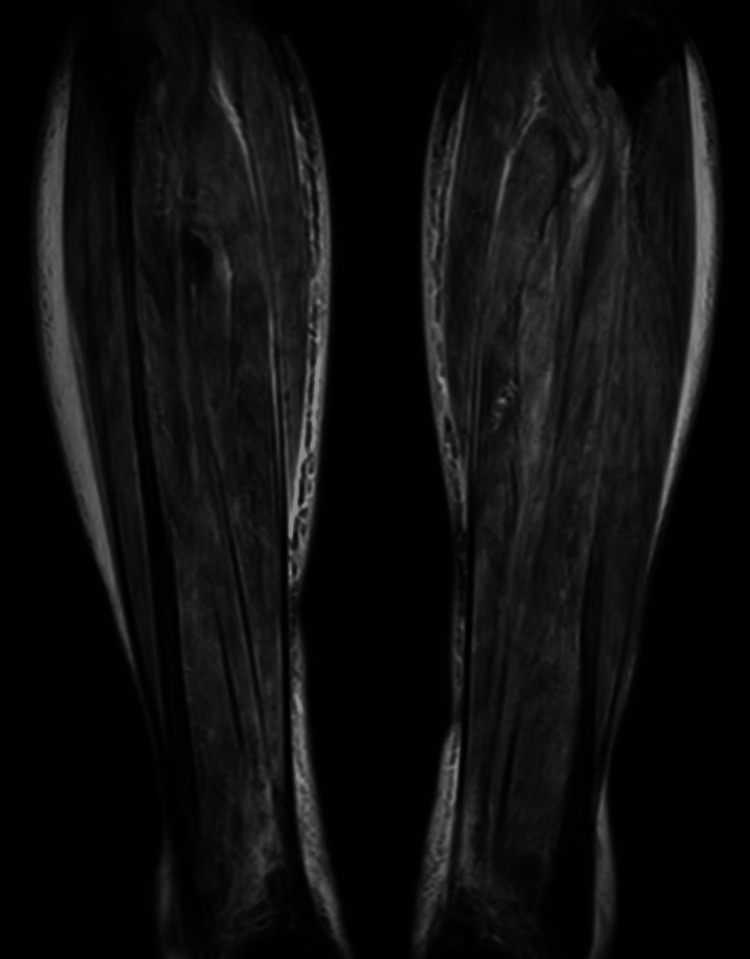
Magnetic resonance imaging of the bilateral triceps surae muscles. Magnetic resonance imaging of the leg revealed high signal intensity in the bilateral triceps surae muscles on T2 fat-saturated images.

**Figure 2:**
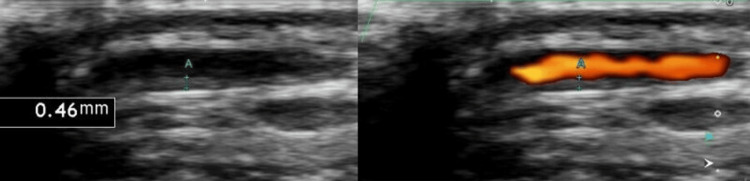
Ultrasonography of the right temporal artery. Ultrasonography of the right temporal artery revealing vessel wall thickening (0.46 mm) and luminal narrowing with power Doppler mode on the right image.

**Figure 3:**
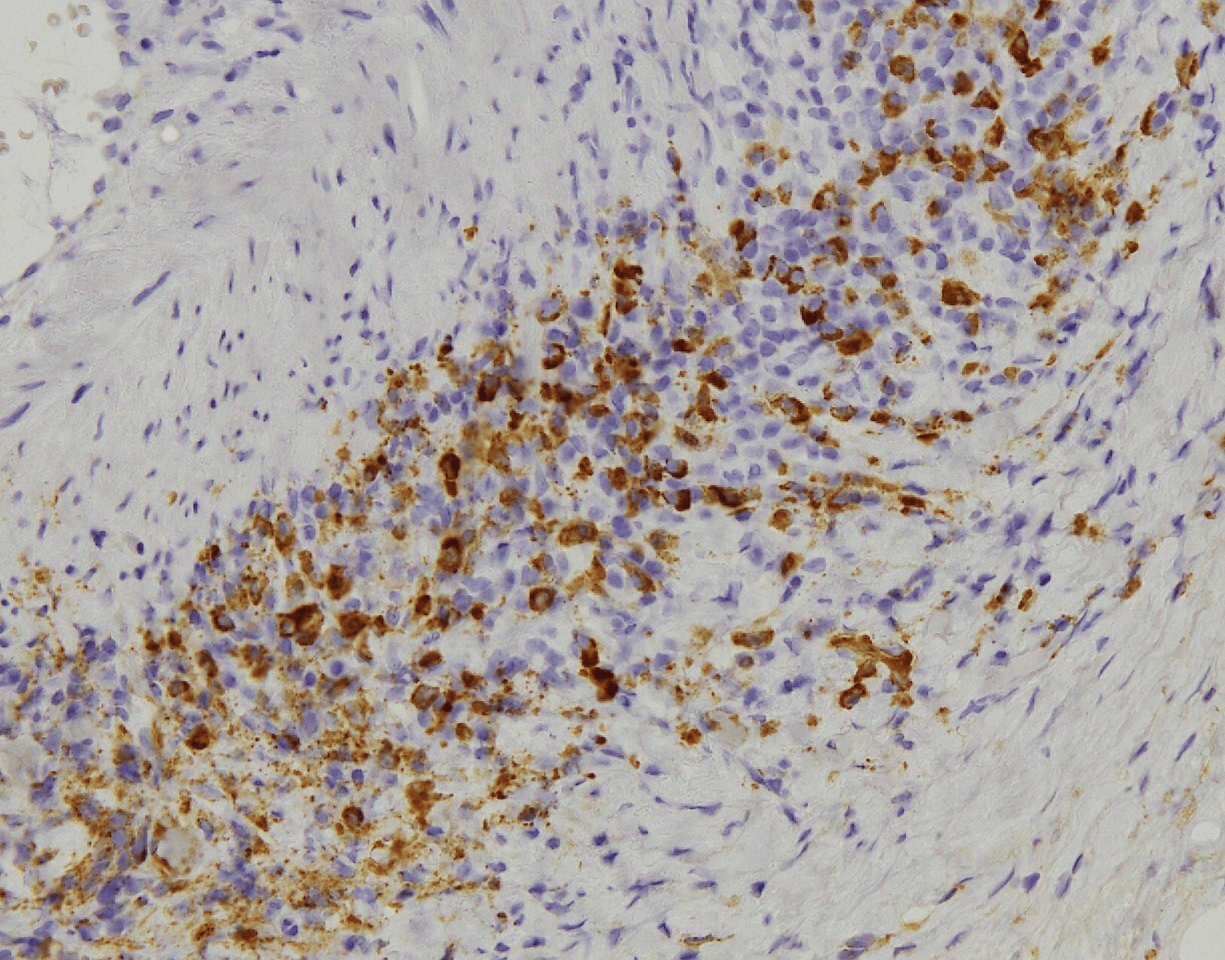
IgG4 immunostaining of the temporal artery. Immunoglobulin G4 (IgG4) immunostaining of the temporal artery showing more than 60 IgG4-positive cells per high-power field (magnification, ×400).

Magnetic resonance imaging of the leg revealed high signal intensity in the bilateral triceps surae muscles on T2 fat-saturated images (Figure 1), and ultrasonography of the right temporal artery revealed vessel wall thickening with luminal narrowing (Figure 2). A biopsy of the right temporal artery was performed, which revealed plasma cell infiltration in the adventitia, with approximately 60 IgG4-positive cells per high-magnification field (Figure 3) and an IgG4/IgG-positive cell ratio of 80%. Storiform fibrosis and obliterative phlebitis were not identified in the biopsy specimen. Additional serum analysis revealed an IgG4 level of 429 mg/dL. Based on these findings, the diagnosis of IgG4-related periarteritis of the temporal artery and IgG4-related myositis was established. Representative organ involvement of IgG4-RD, such as in the lacrimal glands, salivary glands, kidneys, pancreas, retroperitoneum, or periaortic region, was not observed in this case.

Having initiated treatment with prednisolone (30 mg/day), the fever had subsided on the following day, and by the second day, the leg pain and jaw claudication had disappeared. The patient was discharged 25 days after transfer, and prednisolone was gradually tapered.

## Discussion

This case was associated with two notable findings. Firstly, periarteritis of the temporal artery can be caused by IgG4-related arteritis/periarteritis, and, secondly, in cases of fever of unknown origin, assessment of jaw claudication and palpation of the temporal arteries can aid in diagnosis, even in the absence of headache.

IgG4-related arteritis/periarteritis can occur in the temporal artery. Although the aorta is the most common lesion site in IgG4-related arteritis/periarteritis, the carotid, iliac, coronary arteries, and head and neck arteries have also been reported as targets of IgG4-related arteritis/periarteritis [2,7]. However, IgG4-related temporal arteritis/periarteritis is rare, with only four cases reported to date [3-6]. Of these four cases, three had aneurysms of the temporal artery [3-5], whereas the remaining patient had coronary periarteritis and an ascending aortic aneurysm, although no temporal artery aneurysm [6]. Our case was notable for the absence of any aneurysm. In addition to giant cell arteritis (GCA) and IgG4-RD, temporal arteritis caused by anti-neutrophilic cytoplasmic antibody-associated vasculitis and polyarteritis nodosa had been reported [8]. When temporal arteritis is suspected, not only GCA but also IgG4-RD, as well as medium- and small vessel vasculitis, should be considered in the differential diagnosis, and relevant clinical assessment and diagnostic investigations should be performed.

In evaluating a patient with a fever of unknown origin, even without complaints of headache, assessment of jaw claudication and palpation of the temporal arteries can be useful in elucidating the cause of the fever. The sensitivity of headache, jaw claudication, and loss of temporal artery pulsation for the diagnosis of GCA is 72.2%, 37.5%, and 38.2%, respectively, and their specificity is 45.7%, 92.3%, and 88.2% [9], respectively. Therefore, even in the absence of headache, other highly specific symptoms and physical findings, such as jaw claudication and pulsation of the temporal artery, should also be assessed. Although the above data pertain to GCA, our case suggests that similar symptoms and physical findings are useful for diagnosing IgG4-related arteritis/periarteritis. Among the four previously reported cases of IgG4-related temporal arteritis/periarteritis, headache was observed in two cases; however, neither jaw claudication nor loss of temporal artery pulsation was observed [3-6]. Further accumulation of cases is needed to clarify the diagnostic characteristics of symptoms and physical findings in IgG4-related arteritis/periarteritis of the temporal artery.

The diagnostic validity should also be discussed, given the unusual presentation in this case. Although fever is uncommon in IgG4-RD, it has been observed in 8.1% of patients with IgG4-related arteritis/periarteritis and retroperitoneal fibrosis in a cohort study [10]. Additionally, while elevated CRP levels are generally considered atypical in IgG4-RD, they have been reported in cases with IgG4-related myositis [10]. For instance, one report described a patient with a markedly elevated CRP level of 33 mg/dL, suggesting that systemic inflammation may be more pronounced in such cases [11]. These findings highlight that fever and marked inflammation do not necessarily preclude the diagnosis of IgG4-RD.

Histopathological features characteristic of GCA include multinucleated giant cell infiltration, transmural inflammation, and disruption of the internal elastic lamina [12], although none of these findings were observed in the present case. Instead, the biopsy revealed prominent plasma cell infiltration and an increased number of IgG4-positive cells and a high IgG4/IgG-positive cell ratio, which are features characteristic of IgG4-related periarteritis/arteritis. While storiform fibrosis and obliterative phlebitis are considered hallmark features of IgG4-RD, a previous study reported that they were observed in only 64.3% and 57.1% of biopsy specimens, respectively, in cases of IgG4-related periarteritis/arteritis [10]. This suggests that such features may be absent in small biopsy samples, as was likely the case here.

## Conclusions

In summary, we described a case of IgG4-related periarteritis of the temporal artery presenting with fever and jaw claudication. Even if the patient does not complain of headache, assessment for jaw claudication and palpation of the temporal arteries can be useful in determining the cause of fever of unknown origin.

## References

[1] Kamisawa T, Zen Y, Pillai S, Stone JH (2015). IgG4-related disease. Lancet.

[2] Fragoulis GE, Evangelatos G, Tektonidou MG (2021). Vasculitis beyond aortitis in IgG4-related disease (IgG4-RD): case report and review of the literature. Clin Rheumatol.

[3] Ferfar Y, Charlotte F, Cacoub P, Saadoun D (2021). Temporal arteritis in IgG4 related disease. Joint Bone Spine.

[4] Kuma S, Takeshima T, Ohga T, Nozoe T, Sueishi K (2017). Superficial temporal artery aneurysm associated with immunoglobulin G4-related disease. J Vasc Surg Cases Innov Tech.

[5] Kousuke Y, Kengo N, Thutomu M, Mitsuharu N (2017). A case of IgG4-related periarteritis of the left temporal arteries. (Article in Japanese). Jibi To Rinsho.

[6] Kaymakci M, Elfishawi M, Koster MJ, Hurst PD, Warrington KJ (2023). Clinical images: IgG4-related disease: a giant cell arteritis mimic. Arthritis Rheumatol.

[7] Mitani K, Funaki T, Tanji M (2022). Detecting immunoglobulin G4-related intracranial arteriopathy with magnetic resonance vessel wall imaging: a preliminary experience in two cases. BMC Neurol.

[8] Cavazza A, Muratore F, Boiardi L (2014). Inflamed temporal artery: histologic findings in 354 biopsies, with clinical correlations. Am J Surg Pathol.

[9] van der Geest KS, Sandovici M, Brouwer E, Mackie SL (2020). Diagnostic accuracy of symptoms, physical signs, and laboratory tests for giant cell arteritis: a systematic review and meta-analysis. JAMA Intern Med.

[10] Mizushima I, Kasashima S, Fujinaga Y (2019). Clinical and pathological characteristics of IgG4-related periaortitis/periarteritis and retroperitoneal fibrosis diagnosed based on experts’ diagnosis. Ann Vasc Dis.

[11] Casteleyn V, Radbruch H, Diekhoff T, Rose T, Spengler L, Schneider U, Stenzel W (2019). Immunoglobulin (Ig)G-4 related myositis - a new entity?. Neuromuscul Disord.

[12] Muniz Castro HM, Bhattacharjee MB, Chaudhry IA, Chuang AZ, Mankiewicz KA, Adesina OO (2022). Diagnosis of giant cell arteritis using clinical, laboratory, and histopathological findings in patients undergoing temporal artery biopsy. Clin Neurol Neurosurg.

